# First Comprehensive Assessment of Quality of Life in Cancer Patients in Afghanistan: Insights From Herat Regional Hospital

**DOI:** 10.1002/cnr2.70404

**Published:** 2025-11-27

**Authors:** Ali Rahimi, Enayatollah Ejaz, Farooq Ahmad Saddiqi, Mohammad Masudi, Musa Joya, Nasar Ahmad Shayan

**Affiliations:** ^1^ Department of Curative Medicine, Faculty of Medicine Jami University Herat Afghanistan; ^2^ Department of Public Health and Infectious Disease, Faculty of Medicine Herat University Herat Afghanistan; ^3^ Department of Pediatrics, Faculty of Medicine Herat University Herat Afghanistan; ^4^ Department of Oncology Herat Regional Hospital Herat Afghanistan; ^5^ Department of Physics University of Surrey Guildford UK; ^6^ Department of Epidemiology and Biostatistics, Schulich School of Medicine and Dentistry Western University London Ontario Canada

**Keywords:** Afghanistan, cancer, Herat Regional Hospital, quality of life

## Abstract

**Background:**

This study marks the first extensive evaluation of quality of life (QoL) among cancer patients in Afghanistan, carried out at the Oncology Department of Herat Regional Hospital. Its primary objective was to assess the QoL of cancer patients and to determine key factors influencing it, utilizing the validated European Organization for Research and Treatment of Cancer (EORTC) QLQ‐C30 instrument.

**Aims:**

The study aimed to determine overall QoL levels among Afghan cancer patients and to examine the impact of sociodemographic and clinical characteristics, including disease stage and gender differences, on functional and symptom related domains.

**Methods and Results:**

A total of 230 patients diagnosed with cancer participated in this cross‐sectional survey conducted between February and May 2024. Data regarding sociodemographic characteristics and clinical profiles were collected, and QoL outcomes were examined across both functional and symptom‐related domains. The findings indicated that esophageal and gastric cancers were the most common types observed, with the majority of patients presenting at stage two of the disease. Among the functional domains, cognitive functioning received the highest scores, whereas social functioning scored the lowest. Financial hardship emerged as the most burdensome symptom reported. Disease progression, marked by advancing cancer stages, was associated with significant declines in physical and role functioning, along with increases in symptoms such as dyspnea and insomnia. Gender differences were notable, with male patients reporting higher overall QoL compared to female patients. Economic challenges were found to have a considerable negative effect on QoL outcomes.

**Conclusion:**

As a pioneering study in Afghanistan, these results emphasize the pressing need for interventions that address the physical, psychological, and economic hardships faced by cancer patients. The study further stresses the importance of promoting early diagnosis, developing individualized treatment plans, and providing comprehensive supportive care to enhance patient well‐being. These insights offer a foundation for developing cancer care policies and integrating standardized QoL assessment tools within clinical practice in Afghanistan and similar resource‐limited environments. These findings provide crucial evidence for policymakers to develop targeted, gender‐sensitive interventions to mitigate financial toxicity and improve supportive care within Afghanistan's fragile health system.

## 1 Introduction

Afghanistan, a war‐torn and low‐income country in South Central Asia, has faced decades of civil war, climate change, and political instability, significantly impacting its population of over 43 million people [1, 2]. Approximately 97% of Afghans live in poverty, with more than half experiencing extreme poverty, especially women [3]. The collective effects of war, poverty, unemployment, illiteracy, inadequate healthcare, and lack of access to healthy food and clean water have contributed to the prevalence of various diseases. Among these, cancer is emerging as a rapidly growing health issue that remains underdiagnosed and undertreated in Afghanistan.

Cancer statistics in Afghanistan highlight the severity of the issue. According to the World Health Organization (WHO), there were 19 450 new cancer cases and 14 746 cancer‐related deaths reported in 2018, with breast, stomach, oral, esophageal, and lung cancers being the most common [4, 5]. Between 2018 and 2020, 10 066 new cancer cases were recorded, predominantly among men aged 50 years and older, with esophageal and stomach cancers being the most frequent in men and breast cancer in women [6]. By 2020, approximately 24 000 new cancer cases were reported, reflecting a high mortality rate due to limited diagnostic and treatment facilities [4, 6]. These alarming statistics underscore the urgent need for comprehensive cancer care and preventive measures in Afghanistan.

Quality of life (QoL) is recognized as a fundamental aspect of healthcare, especially in the context of chronic conditions such as cancer [7]. According to the WHO, QoL reflects an individual's perceived life satisfaction and overall state of well‐being [8]. A cancer diagnosis, along with its associated treatments, can profoundly impair QoL by negatively influencing physical, emotional, social, and functional domains [9]. Therefore, evaluating QoL becomes a crucial component of clinical decision‐making, particularly in later stages of cancer, where the emphasis often shifts toward improving patient comfort and life satisfaction [10]. Incorporating QoL measurements into routine medical care is essential for delivering comprehensive, patient‐centered care that reflects individual preferences and priorities.

The conceptual framework for this study is grounded in the multidimensional model of QoL operationalized by the EORTC QLQ‐C30 [11]. This instrument assesses QoL across five core functional domains [12, 13]: physical functioning (ability to perform daily activities), role functioning (ability to work or pursue hobbies), emotional functioning (presence of anxiety or depression), cognitive functioning (concentration and memory), and social functioning (impact of health on social activities). It complements this with an assessment of symptom burden through scales for fatigue, pain, and nausea/vomiting, as well as single items for other common symptoms and the perceived financial impact of the disease. A global health status/QoL scale provides an overall patient self‐rating of well‐being. This comprehensive structure allows for a nuanced understanding of how cancer impacts a patient's life beyond purely clinical metrics, making it a cornerstone of patient‐centered oncology research worldwide.

The National Cancer Control Program in Afghanistan, established in 2016, has made strides in addressing cancer care. The program's initiatives include prevention, early diagnosis, treatment, supportive care, and surveillance, as well as the establishment of cancer centers in Kabul, Mazar, and Herat to improve access to services [14]. The cancer center at Herat Regional Hospital, inaugurated in 2018, represents a significant milestone in providing specialized care for cancer patients in western Afghanistan. Despite these advancements, the mortality rate remains high due to inadequate resources, highlighting the importance of addressing both clinical and QoL aspects in cancer care.

While data from Afghanistan is nonexistent, studies from other low‐ and middle‐income countries (LMICs) in Asia provide a valuable comparative context. Research on cancer patients in South and East Asia consistently highlights that, beyond clinical symptoms, sociodemographic factors such as low income, lower educational attainment, and unemployment are powerful determinants of poorer QoL [15]. Financial toxicity, in particular, emerges as a dominant and distressing theme, often forcing patients to abandon treatment due to catastrophic out‐of‐pocket (OOP) expenditures. Furthermore, studies frequently report significant burdens of fatigue and emotional distress, with women often experiencing a lower overall QoL compared to men, a finding attributed to both clinical and sociocultural factors [16]. These findings from other resource‐constrained Asian settings underscore the urgent need to investigate the specific challenges faced by Afghan patients, whose circumstances are further compounded by decades of conflict, political instability, and a uniquely fragile healthcare system [17].

Internationally, the evaluation of QoL has been instrumental in capturing the effects of cancer and its treatments on patients' daily functioning and overall well‐being. Instruments like the European Organization for Research and Treatment of Cancer's QLQ‐C30 questionnaire have demonstrated strong validity and sensitivity in assessing QoL among cancer populations [18, 19]. These patient‐centered tools offer valuable data on patient‐reported outcomes, informing clinical practice and enhancing the quality of care. Despite this global progress, there has been no thorough investigation into the QoL of cancer patients within Afghanistan. This study represents the first systematic effort to assess QoL among cancer patients at Herat Regional Hospital using a validated assessment tool, with the goal of identifying the key determinants that influence patient well‐being.

The findings from this pioneering research will be instrumental in shaping effective therapeutic interventions, guiding clinical decisions, and informing health policies to enhance cancer care in Afghanistan. Additionally, this study will contribute to the global understanding of QOL in cancer patients, particularly in low‐income and conflict‐affected settings. By addressing these critical gaps, this research seeks to improve patient outcomes and standardize cancer care in Afghanistan and similar low‐resource contexts.

## 2 Methods

### 2.1 Study Design, Setting, and Period

This cross‐sectional survey took place in the Oncology Department of Herat Regional Hospital, Herat, Afghanistan, from February 1, 2024 to May 1, 2024.

### 2.2 Sample Size Calculation

The minimum required sample size was calculated to be 223, based on a 99% confidence level, an assumed population proportion (*p*) of 0.031, and a margin of error (*e*) of 3%. The formula used for this calculation was *n* = Z^2^(*p* × *q*)/*E* ^2^, where *p* = 0.031, *q* = 1 − *p* (0.969), *Z* = 2.576 corresponding to the 99% CI, and *E* = 0.03. To strengthen the study's representativeness and account for possible participant variability, an additional 3% was added to the estimated sample size. Ultimately, data were collected from 230 participants.

### 2.3 Study Tool

The study utilized the EORTC QLQ‐C30 questionnaire, which was employed with official authorization. This instrument consists of 30 items covering 15 distinct domains: five functional domains (physical, role, cognitive, emotional, and social functioning) and nine symptom domains (fatigue, pain, nausea and vomiting, dyspnea, sleep problems, appetite loss, constipation, diarrhea, and financial issues). Additionally, it assesses global health status and overall QOL [19]. Higher scores on the functioning and global health dimensions reflect better QOL, whereas higher scores on symptom scales denote greater symptom severity [11].

### 2.4 Questionnaire Translation, Adaptation, and Pilot Study

Given that the EORTC QLQ‐C30 had not been previously validated in Afghanistan, a rigorous adaptation process was undertaken. The official English version of the EORTC QLQ‐C30 (version 3.0) was translated into Dari, the predominant Persian dialect spoken in Herat, by two independent bilingual health professionals. A consensus version was developed and subsequently back‐translated into English by a third independent translator, who had no prior knowledge of the original questionnaire, to ensure conceptual and semantic equivalence. This pre‐final Dari version was reviewed by a local oncologist and two cancer patients for clarity, cultural appropriateness, and ease of understanding.

Prior to the main study, a pilot study was conducted with 30 cancer patients at Herat Regional Hospital who met the main study's inclusion criteria but were not included in the final sample. The purpose of the pilot was to assess the acceptability of the questionnaire, the clarity of the items, and the time required for completion, and to establish the internal consistency (reliability) of the translated instrument. The questionnaire was well accepted by all pilot participants, with an average completion time of 15 min. The internal consistency of the multi‐item scales of the Dari version of the EORTC QLQ‐C30 was assessed using Cronbach's alpha coefficient. An alpha value of ≥ 0.70 was considered indicative of acceptable reliability for each of the dimensions of the scale.

### 2.5 Data Collection

Data collection was scheduled twice weekly, on Mondays and Wednesdays, corresponding with the researchers' clinical schedules. Interviews were conducted by author E.E. Before participation, patients were briefed about the study, and written informed consent was obtained. Eligible participants included all adults aged 18–76 who either visited the outpatient clinic or were newly hospitalized during these days. Literate individuals self‐administered the questionnaire, while illiterate participants received assistance, with the researchers reading the questions aloud and recording the answers. Exclusion criteria included previous study participants, individuals unable to provide informed consent (such as unconscious patients), patients with unconfirmed cancer diagnoses, and minors under 18 years. Sociodemographic details (age, sex, marital status, religious affiliation, socioeconomic status, and educational background) along with clinical characteristics (primary tumor site, stage of disease, and treatment modality) were documented.

### 2.6 Data Analysis

The collected data were first entered into Microsoft Excel and subsequently analyzed using SPSS version 26.0 (IBM Corp., Armonk, NY, USA). Descriptive statistics, including means, standard deviations, frequencies, and percentages, were utilized to summarize variables such as cancer type, disease stage, and treatments. Scores for each EORTC QLQ‐C30 scale were calculated as per the official EORTC scoring manual. Raw scores were linearly transformed to a 0–100 scale [11]. For functional and global QoL scales, higher scores represent a better level of functioning or QoL. Conversely, for the symptom scales and single items (including financial difficulties), higher scores indicate a greater symptom burden or more severe problems. Internal consistency of the questionnaire scales was assessed using Cronbach's alpha. Analysis of categorical variables was performed using ANOVA, with statistical significance set at a *p*‐value of ≤ 0.05.

### 2.7 Ethical Considerations

The study was conducted at the Oncology Unit of Herat Regional Hospital and received ethical approval from the Institutional Review Board of Jami University, which oversees research ethics and issues approval codes for registered proposals. Ethical clearance for this study was granted on January 27, 2024 (Ref: J.2024.1.27.1). All participants provided written informed consent prior to participation. For participants who were illiterate, informed consent was obtained through their legally authorized representatives (LARs). Participant confidentiality and privacy were maintained throughout the study in accordance with the Declaration of Helsinki and ethical principles guiding research involving human subjects.

## 3 Results

A total of 230 individuals diagnosed with cancer participated in this study. Of these, 125 were male, comprising 54.3% of the sample population. The age of participants spanned from 18 to over 70 years, with the majority falling within the 50–59‐year age range (30.9%). Most were married (85.2%) and identified as Tajik^1^ (41.7%). Half of the participants reside in Herat (49.6%) and their illiteracy rate was 83.9%. The largest group was self‐employed (40.4%). In addition, most of the participants declared a poor economic status (89.1%) (Table 1).

**TABLE 1:** Sociodemographic characteristics of the participants (Herat, Afghanistan in 2024).

	*N*	%
Gender		
Male	125	54.3
Female	105	45.7
Marital status		
Single	16	7.0
Married	196	85.2
Divorced	0	0.0
Widow	18	7.8
Age range (years)		
18–39	46	20.0
40–49	44	19.1
50–59	71	30.9
60–69	54	23.5
> 70	15	6.5
Ethnicity		
Pashton	88	38.3
Tajik	96	41.7
Hazara	26	11.3
Ozbek	9	3.9
Aimaq	11	4.8
Address		
Herat	114	49.6
Farah	31	13.5
Badghis	25	10.9
Others	60	26.1
Level of education		
Illiterate	193	83.9
Primary	32	13.9
University	5	2.2
Employment		
Not working	57	24.8
Housewife	80	34.8
Self‐employed	93	40.4
Economic status		
Good	7	3.0
Average	18	7.8
Poor	205	89.1
Total	230	100.0

Approximately 80% of the participants had received their cancer diagnosis within the preceding year. Esophageal cancer emerged as the most prevalent type, followed by stomach cancer. The majority of cases were classified as stage two disease. Chemotherapy was the most common treatment modality administered over the past 6 months. A comprehensive breakdown of the cancer types, stages, and treatment methods is presented in Table 2.

**TABLE 2:** Cancer‐related characteristics of the patient.

	Gender					
	Male		Female		Total	
	*N*	%	*N*	%	*N*	%
Primary diagnosis						
Lip, oral cavity = C00–06	2	1.6	1	1.0	3	1.3
Nasopharynx = C11	4	3.2	2	1.9	6	2.6
Hypopharynx = C12–13	2	1.6	0	0.0	2	0.9
Esophagus = C15	35	28.0	33	31.4	68	29.6
Stomach = C16	26	20.8	4	3.8	30	13.0
Colorectum = C18–C21	10	8.0	6	5.7	16	7.0
Liver and intrahepatic bile ducts = C22	4	3.2	2	1.9	6	2.6
Gallbladder = C23	1	0.8	3	2.9	4	1.7
Pancreas = C25	3	2.4	0	0.0	3	1.3
Trachea, bronchus, and lung = C33–34	2	1.6	4	3.8	6	2.6
Melanoma of skin = C43	3	2.4	1	1.0	4	1.7
Mesothelioma = C45	1	0.8	0	0.0	1	0.4
Kaposi sarcoma = C46	2	1.6	2	1.9	4	1.7
Breast = C50	0	0.0	29	27.6	29	12.6
Vulva = C51	0	0.0	1	1.0	1	0.4
Vagina = C52	0	0.0	2	1.9	2	0.9
Cervix uteri = C53	0	0.0	3	2.9	3	1.3
Corpus uteri = C54	0	0.0	1	1.0	1	0.4
Ovary = C56	1	0.8	2	1.9	3	1.3
Prostate = C61	3	2.4	0	0.0	3	1.3
Testis = C62	2	1.6	0	0.0	2	0.9
Kidney = C64	2	1.6	2	1.9	4	1.7
Brain, central nervous system = C70–72	4	3.2	0	0.0	4	1.7
Thyroid = C73	1	0.8	3	2.9	4	1.7
Hodgkin lymphoma = C81	3	2.4	2	1.9	5	2.2
Non‐Hodgkin lymphoma = C82–86 + C88	6	4.8	1	1.0	7	3.0
Leukemia = C91–95	3	2.4	0	0.0	3	1.3
Retinoblastoma = C69.20	1	0.8	0	0.0	1	0.4
Lumbar sarcoma = C41.2	1	0.8	0	0.0	1	0.4
Eye sarcoma = C49.9	1	0.8	1	1.0	2	0.9
Osteosarcoma = C41.9	2	1.6	0	0.0	2	0.9
Time of diagnosis						
Less than 12 months	52	41.6	33	31.4	85	37.0
13–24 months	53	42.4	39	37.1	92	40.0
25–36 months	11	8.8	19	18.1	30	13.0
37–48 months	2	1.6	4	3.8	6	2.6
49–60 months	7	5.6	8	7.6	15	6.5
> 60 months	0	0.0	2	1.9	2	0.9
Unknown	0	0.0	0	0.0	0	0.0
Cancer stage						
Stage one	48	38.4	21	20.0	69	30.0
Stage two	62	49.6	52	49.5	114	49.6
Stage three	10	8.0	27	25.7	37	16.1
Stage four	5	4.0	5	4.8	10	4.3
Type of intervention during the past 6 months						
Surgery	5	4.0	0	0.0	5	2.2
Chemotherapy	75	60.0	50	47.6	125	54.3
Radiology	0	0.0	1	1.0	1	0.4
Surgical + Chemotherapy	39	31.2	47	44.8	86	37.4
Surgical + Radiology	2	1.6	6	5.7	8	3.5
No treatment	4	3.2	1	1.0	5	2.2

Most patients reported that they were looked after by a first‐degree relative in the past 30 days, with only a small percentage receiving professional home healthcare services. Table 3 shows the details of home care services during this period (Table 3).

**TABLE 3:** Home care uses during the previous 30 days.

	Gender					
	Male		Female		Total	
	*N*	%	*N*	%	*N*	%
Home care provider						
First‐degree relative	86	68.8	82	78.1	168	73.0
Other relative/friend	36	28.8	21	20.0	57	24.8
Other caregivers	3	2.4	2	1.9	5	2.2
Professional home healthcare						
Received	18	14.4	12	11.4	30	13.0
Not received	107	85.6	93	88.6	200	87.0

In the analysis of QOL domains, the cognitive function scale recorded the highest mean score (50.94), while social function scored the lowest (6.03). Among the symptom scales, financial difficulties received the highest score (98.70), indicating a major burden, whereas diarrhea scored the lowest (21.59). Men aged 40–49 years attained the highest cognitive function scores (57.41), and women aged 18–39 years followed closely (55.56). Regarding symptom burden, women aged 40–49 years reported the most severe nausea and vomiting (60.26), while appetite loss was most pronounced among women aged 50–59 years (83.33). The mean score for global health status and overall QOL was 8.73, with men reporting higher scores compared to women (10.60 vs. 8.73) (Table 4).

**TABLE 4:** EORTC QLQ‐C30 reference values for the study population by age and sex.

	Age range (years)										Total			
	18–39		40–49		50–59		60–69		> 70		By gender		Overall	
	Mean	SD	Mean	SD	Mean	SD	Mean	SD	Mean	SD	Mean	SD	Mean	SD
Physical function														
Male	29.29	20.64	32.59	24.19	33.33	18.99	34.37	16.94	31.67	22.04	32.43	19.75	28.64	18.70
Female	32.22	17.15	25.64	12.75	24.26	17.25	17.58	15.33	8.89	15.40	24.13	16.34		
Role function														
Male	15.48	19.74	19.44	15.39	18.57	16.05	22.40	16.73	15.28	15.01	18.67	16.88	16.23	16.62
Female	19.44	16.42	14.74	15.15	13.43	17.28	6.82	12.24	11.11	19.25	13.33	15.91		
Emotional function														
Male	38.99	20.17	31.48	19.50	42.38	25.19	31.77	16.18	25.00	16.67	35.67	20.92	33.51	18.99
Female	28.24	10.36	31.73	18.41	35.65	17.77	24.62	12.46	30.56	17.35	30.95	16.13		
Cognitive function														
Male	55.36	26.47	57.41	23.02	52.38	23.27	49.48	15.55	41.67	18.12	52.00	21.96	50.94	22.03
Female	55.56	21.39	48.72	18.21	53.70	25.23	38.64	18.82	55.56	25.46	49.68	22.17		
Social function														
Male	6.55	13.10	14.81	21.30	8.57	14.78	10.94	15.03	1.39	4.81	8.93	15.20	7.61	15.14
Female	6.48	12.96	7.05	21.17	4.63	12.35	7.58	13.34	0.00	0.00	6.03	15.00		
Fatigue														
Male	80.95	21.57	86.42	11.78	85.08	13.73	80.90	18.77	76.85	20.90	82.49	17.53	83.82	16.98
Female	80.25	18.50	84.19	18.64	87.65	15.21	87.37	13.84	85.19	6.42	85.40	16.26		
Nausea/vomiting														
Male	22.02	23.59	33.33	31.83	54.76	38.26	53.65	28.94	36.11	36.81	42.27	34.30	45.72	32.11
Female	35.19	30.19	60.26	27.11	49.54	29.94	51.52	25.15	38.89	25.46	49.84	28.91		
Pain														
Male	79.76	17.78	68.52	19.71	75.71	11.68	70.83	20.30	84.72	16.60	75.20	17.66	77.25	17.67
Female	76.85	19.92	82.69	14.51	75.00	18.90	87.88	12.79	66.67	16.67	79.68	17.45		
Dyspnea														
Male	22.62	36.35	18.52	26.13	28.57	32.48	28.13	26.92	30.56	33.21	25.87	31.07	33.04	32.07
Female	33.33	36.16	47.44	26.95	37.96	32.02	43.94	31.52	66.67	0.00	41.59	31.28		
Insomnia														
Male	57.14	34.97	57.41	31.94	61.90	26.99	56.25	26.01	69.44	30.01	59.47	29.51	63.91	30.00
Female	57.41	31.94	82.05	23.53	61.11	29.28	80.30	28.47	44.44	19.25	69.21	29.85		
Appetite loss														
Male	53.57	33.13	59.26	33.44	76.19	28.66	70.83	30.23	50.00	38.92	64.80	32.87	69.42	30.48
Female	64.81	31.25	76.92	22.65	74.07	25.34	83.33	26.73	66.67	33.33	74.92	26.47		
Constipation														
Male	38.10	38.18	55.56	39.61	47.62	37.30	56.25	38.28	61.11	39.78	50.13	38.48	50.29	37.35
Female	31.48	35.19	53.85	35.37	52.78	35.97	59.09	35.53	44.44	38.49	50.48	36.14		
Diarrhea														
Male	22.62	27.30	31.48	37.00	18.10	27.23	30.21	34.24	22.22	29.59	24.53	30.86	23.19	29.11
Female	16.67	28.58	24.36	24.14	18.52	26.96	28.79	29.63	11.11	19.25	21.59	26.95		
Financial problems														
Male	100.00	0.00	94.44	12.78	100.00	0.00	98.96	5.89	100.00	0.00	98.93	5.89	98.70	8.43
Female	98.15	7.86	96.15	19.61	99.07	5.56	100.00	0.00	100.00	0.00	98.41	10.72		
Global health status/QoL														
Male	9.23	11.42	9.26	11.39	10.00	9.22	13.02	10.14	11.11	10.26	10.60	10.34	9.75	10.85
Female	13.43	15.95	11.22	11.04	6.48	7.74	5.30	11.37	11.11	12.73	8.73	11.40		

Assessment of QOL using the EORTC QLQ‐C30 indicated that functioning scale scores declined with increasing cancer stage. For symptom scales, fatigue, nausea, vomiting, and pain were initially more pronounced at earlier cancer stages but tended to lessen in severity as the disease progressed. In contrast, dyspnea and insomnia showed worsening patterns as cancer advanced (Table 5). The ANOVA test was used to compare mean differences across cancer stages. Significant findings were observed in several areas: physical functioning scores decreased significantly with disease progression (*F*(3,226) = 9.448, *p* < 0.001); role functioning also showed a notable decline (*F*(3,226) = 4.316, *p* = 0.006); dyspnea severity increased (*F*(3,226) = 5.227, *p* = 0.002); constipation scores varied significantly (*F*(3,226) = 3.673, *p* = 0.013); and global health status/QOL decreased markedly (*F*(3,226) = 3.690, *p* = 0.013). No statistically significant differences were found across stages for emotional functioning, cognitive functioning, social functioning, fatigue, nausea/vomiting, pain, insomnia, appetite loss, diarrhea, or financial burden, with all *p*‐values exceeding 0.05.

**TABLE 5:** EORTC QLQ‐C30 scores according to cancer stage.

	Cancer stage									
	Stage one		Stage two		Stage three		Stage four		ANOVA	
	Mean	SD	Mean	SD	Mean	SD	Mean	SD	*F*	*p*
Physical function	36.23	19.32	28.25	16.95	18.92	17.32	16.67	16.70	9.448	< 0.001
Role function	21.01	16.33	15.79	17.01	11.26	14.19	6.67	14.05	4.316	0.006
Emotional function	34.06	19.05	35.31	18.89	29.28	18.49	25.00	19.64	1.655	0.177
Cognitive function	52.66	20.73	52.19	22.23	48.65	22.35	33.33	22.22	2.576	0.055
Social function	10.87	16.87	6.29	12.62	6.76	19.03	3.33	10.54	1.675	0.173
Fatigue	79.23	18.66	85.58	15.50	86.19	16.85	86.67	17.99	2.467	0.063
Nausea/vomiting	45.17	33.95	46.64	30.42	46.85	33.76	35.00	34.65	0.421	0.738
Pain	74.15	17.97	77.05	17.23	81.98	16.38	83.33	22.22	2.018	0.112
Dyspnea	22.22	27.81	35.09	30.99	41.44	33.71	53.33	45.00	5.227	0.002
Insomnia	57.97	30.06	64.33	29.66	72.97	28.15	66.67	35.14	2.093	0.102
Appetite loss	62.32	33.78	73.68	27.51	71.17	28.50	63.33	39.91	2.200	0.089
Constipation	44.93	38.27	57.02	35.95	46.85	35.54	23.33	38.65	3.673	0.013
Diarrhea	18.36	26.53	26.90	30.36	18.92	24.27	30.00	42.89	1.715	0.165
Financial problems	98.07	7.85	99.42	4.40	97.30	16.44	100.00	0.00	0.822	0.483
Global health status/QoL	11.84	12.16	10.31	10.59	5.41	7.91	5.00	8.96	3.690	0.013

## 4 Discussion

This study provides the first systematic assessment of QoL among cancer patients in Afghanistan, a nation where the clinical realities of a cancer diagnosis are inseparable from the compounded crises of prolonged conflict, economic collapse, and a deeply fractured healthcare system. Our findings reveal a population suffering from profound functional impairments and a high symptom burden, with outcomes deeply intertwined with the country's unique sociocultural and political landscape. This discussion is organized thematically to interpret our quantitative findings through the lens of these contextual realities, tracing the data back to the lived experiences of patients navigating care in one of the world's most challenging settings.

### 4.1 Financial Toxicity as the Dominant Symptom in a Collapsed Economy

The most striking finding of this study is the near‐universal mean score for “financial difficulties” (98.70), making it the most severe symptom reported by a significant margin. This score is not merely a reflection of individual hardship but a direct measure of a systemic collapse. Afghanistan is a low‐income country where approximately 97% of the population lives in poverty [17]. Decades of war have devastated the economy, and the healthcare system is critically underfunded, heavily reliant on dwindling international aid, and unable to provide a safety net for its citizens [21]. Moreover, rare and complex cases mostly have to be handled in private hospitals due to the lack of adequate equipment and specialized services in public facilities [22, 23]. Consequently, the financial burden of a complex illness like cancer falls almost entirely on the patient and their family, leading to catastrophic expenditure.

This extreme financial toxicity is exacerbated by a “hidden” economy of OOP payments for services that the public system cannot provide. Even in major government cancer centers, essential diagnostic tools such as CT scans, MRIs, and mammography are often unavailable, forcing patients to seek these services at private facilities at a high cost [24]. One study found that while the government covers a large portion of treatment costs, the 14% paid OOP by patients for diagnostics and other services represents an insurmountable sum for a population where many struggle to afford food [25]. This burden drives a phenomenon of “cross‐border cancer migration,” where patients who can gather the resources undertake arduous journeys to neighboring countries like Pakistan for care. This process creates a new layer of economic hardship, including visa fees, travel, and accommodation costs, and frequently results in patients becoming lost to follow‐up, further compromising their outcomes. The near‐perfect score for financial difficulty in our study is thus a quantitative echo of this desperate reality.

Additionally, the socioeconomic status and cultural background of the patients in our study significantly influenced their QoL. The majority of participants reported poor economic status, which is similar to the findings of Jacob et al., who emphasized the critical role of socioeconomic factors in determining QoL among patients with advanced cancers [26]. The high financial burden and associated stress level highlight the importance of addressing these issues to improve patient QoL. This is further supported by Nolte et al., who emphasized the importance of considering cultural contexts in QoL assessments [27]. Our findings suggest that interventions aimed at improving QoL in cancer patients must address socioeconomic disparities and cultural differences as these factors significantly impact patients' health outcomes.

### 4.2 The Weight of Gender: Unpacking Disparities in a Restrictive Environment

Our study revealed a significant gender disparity, with men reporting a higher overall QoL than women (10.60 vs. 8.73). This finding is a direct reflection of the deeply entrenched gender inequality that defines Afghan society, a country that ranks last globally in the Gender Gap Index [28]. Women face systemic discrimination, including severe restrictions on their movement, education, and employment, which have intensified since August 2021. A cancer diagnosis, therefore, places an additional, crushing weight on a population already marginalized and disempowered [21].

This disparity is not abstract; it is a clinical problem rooted in specific, gendered barriers to care. Cultural norms and official policies often prevent women from being treated by male physicians [29]. However, Afghanistan faces a dire shortage of female healthcare workers—a crisis guaranteed to worsen due to the ban on higher education for women, which has blocked the training of future female doctors and nurses [30]. Furthermore, the requirement for a *mahram* (women's near male family member) to accompany women for travel makes accessing centralized health facilities a logistical and financial impossibility for many, particularly widows or those whose male relatives must work [21, 28]. These barriers delay diagnosis and interrupt treatment, leading to the worse symptom burdens—such as nausea, vomiting, and appetite loss—observed among women in our study. The extremely low mean score for social functioning (6.03) across all patients is particularly acute for women. Already restricted in their public lives, a cancer diagnosis can sever their limited social ties, transforming a medical condition into a state of near‐total social seclusion, which the QLQ‐C30 instrument captures as a rock‐bottom score.

Furthermore, our study revealed that men reported higher overall QoL function scores than women which is consistent with Mols et al. study findings. They found significant sex‐related differences in QoL scores among cancer patients [31]. The lower QoL scores in women may be attributed to the higher burden of symptoms, like nausea, vomiting, and appetite loss, as documented by McLachlan et al. [32]. Additionally, gender‐specific differences in emotional and cognitive functions, as highlighted by Coates et al., suggest that psychosocial factors may differentially impact men and women [33]. This underscores the need for tailored interventions that consider these differences to effectively manage the QoL of both male and female cancer patients.

### 4.3 Impaired Functioning Within a Fractured Care System

The observed significant decline in physical and role functioning with advancing cancer stage is an expected clinical finding. However, in the Afghan context, this decline is actively accelerated by the structure of the healthcare system itself. Decades of conflict have destroyed health infrastructure, and specialized care like oncology is concentrated in a few urban centers such as Herat and Kabul [34]. For the majority of the population living in rural and remote areas, accessing this care is a monumental undertaking.

Patients often endure multiday journeys, sometimes by donkey and then by car, over treacherous terrain to reach a hospital. This physically grueling journey, undertaken by an already frail individual, actively depletes their physiological reserves and contributes directly to the decline in physical functioning measured by our study. The healthcare system, in its inaccessibility, becomes a source of physical burden. Similarly, the precipitous drop in role functioning reflects the complete disruption of life required to seek treatment. In a country with high rates of vulnerable employment, a diagnosis requiring travel to a distant hospital necessitates a complete cessation of work for both the patient and their family caregiver [35]. The time and resources consumed by travel and waiting for a hospital bed—sometimes for days in the open—make maintaining any semblance of a normal life role impossible [36]. The loss of role is not just a consequence of being sick; it is a direct consequence of how and where care is delivered in a fractured system.

### 4.4 Sociocultural Barriers, Stigma, and the Culture of Late‐Stage Presentation

While the majority of patients in our study presented at stage two, this likely reflects a progression from earlier, undiagnosed stages. Studies of Afghan patients who manage to seek care abroad report that over 56% present with advanced (stage three or four) disease, suggesting a widespread culture of late presentation [37]. This pattern is a direct outcome of prevailing sociocultural norms that delay or prevent early help‐seeking. Health‐related decisions are heavily influenced by low health literacy, fatalistic beliefs, and a reliance on family elders. A cancer diagnosis is often viewed as a death sentence, shrouded in stigma and taboo, which leads to fear, denial, and a delay in visiting a health facility until symptoms become unbearable [38]. By this point, the disease is more advanced, treatment options are more limited, and QoL is already severely compromised.

The high rate of illiteracy in our sample (83.9%) is a major structural barrier that perpetuates this cycle. Standard public health campaigns that rely on written materials are ineffective for the vast majority of the population. Health information must be transmitted through channels like radio or community health workers, but outreach is limited and inconsistent [39]. This information vacuum allows misinformation and stigma to flourish, directly contributing to the patterns of delayed diagnosis that underpin many of the poor QoL outcomes documented in this study.

### 4.5 Functional Scales and Cancer Progression

The decline in physical and role functioning with advancing cancer stages observed in our study supports earlier research findings. Aaronson et al. demonstrated that these scales are sensitive to changes in clinical status, indicating that physical deterioration and reduced ability to perform daily activities significantly impact overall QoL in cancer patients [19]. Kaasa et al. found similar results in patients undergoing palliative radiotherapy, where QoL measurements effectively tracked the impact of treatment over time [40].

Moreover, McKernan et al. highlighted the association between QoL and survival in patients with gastroesophageal cancer, emphasizing that physical and role functioning are significant predictors of cancer survival [41]. Our study reinforces these findings, demonstrating that, as cancer progresses, the decline in physical and role functioning is significant, impacting patients' ability to perform daily activities and their overall health.

### 4.6 Symptom Scales and Treatment Side Effects

Symptoms such as dyspnea and insomnia were found to worsen with advancing cancer stages in our study, consistent with the findings of Bergman et al., who reported similar patterns in patients with non‐resectable lung cancer [42]. This suggests that the cumulative burden of symptoms significantly diminishes patients' QoL as cancer progresses. Furthermore, financial problems were prominently reported, echoing the findings of Smith et al., who emphasized the economic weight associated with cancer treatment and its effect on QoL [43].

The high financial burden on the patients in our study is particularly concerning. This aligns with observations by Jayasekara et al., who noted that financial difficulties are a significant predictor of lower QoL in patients with cancer in South Asia [44]. These results underline the need for supportive care strategies that address these financial issues to improve patients' overall QoL.

### 4.7 Impact of Treatment Type

Chemotherapy was the most frequent intervention among our participants, and its side effects were prominently reflected in the symptom scales. Tomaszewski et al. validated the QLQ‐C30 and its gasteroesophageal module in Polish patients and found that treatment side effects significantly impacted QoL [45]. This underscores the necessity of supportive care strategies that mitigate these side effects, thereby enhancing patients' QoL.

McKernan et al. highlighted the association between QoL and survival in patients with gastroesophageal cancer, emphasizing the impact of symptoms for example appetite loss on patient treatment outcomes [41]. This finding is similar to our study results, in which significant correlations between treatment type and symptom burden were observed.

### 4.8 Recommendations for Future Research

More studies are needed to understand the impact of cultural and socioeconomic factors on QoL to develop more effective, economical, and culturally sensitive interventions. In addition, a more specific validation study of the QLQ‐C30 in Afghanistan should be conducted in future studies. Given the context of Afghanistan where this study was conducted, future research should focus on the specific challenges faced by Afghan cancer patients. This includes exploring the effects of conflict and instability on QoL, accessibility to healthcare services, and availability of cancer diagnosis and treatments. Studies should also investigate the role of cultural perceptions and stigmas associated with cancer, which may affect patients' willingness to seek timely medical help. Additionally, evaluating the impact of socioeconomic factors such as poverty and educational status on QoL can help identify critical areas for intervention. Implementing longitudinal studies to track changes in QoL over time, especially in response to different treatments and cancer stages, would provide valuable insights. Collaborating with local healthcare providers and policymakers to improve cancer care infrastructure and accessibility can ensure that the research findings translate into meaningful improvements in patient outcomes.

### 4.9 Limitations and Strengths

This study has several limitations. Its cross‐sectional design precludes the establishment of causal relationships between the observed variables. As a single‐center study conducted in Herat, the findings may not be generalizable to all cancer patients in Afghanistan, who may face different challenges based on geographic location and ethnicity. A major limitation of this study is the absence of a qualitative component. While our quantitative data provide a stark overview of the challenges faced by patients, it cannot capture the rich, explanatory narratives of their lived experiences. In‐depth interviews could have provided crucial insights into how patients and their families navigate the complex interplay of financial toxicity, sociocultural barriers, and a failing health system. For instance, qualitative data could have illuminated the decision‐making processes that lead to catastrophic expenditure or treatment abandonment, and the personal toll of navigating Taliban restrictions on women's mobility. Future research must prioritize a mixed‐methods approach to build upon this foundational study and develop a more holistic understanding of the cancer burden in Afghanistan.

Despite these limitations, the study has notable strengths. It is the first of its kind in Afghanistan, providing a crucial baseline of evidence in a critically under‐researched area. The use of the rigorously translated and pilot‐tested EORTC QLQ‐C30 ensures that the data collected are reliable and valid. By including patients with diverse cancer types and stages, the study offers a broad overview of the challenges faced by this population and identifies clear targets for intervention, such as financial support and symptom management, which can guide the development of future health policies and clinical programs.

### 4.10 Policy Significance in Afghanistan

The findings of this study carry urgent policy implications for Afghanistan's national health strategy and for the international organizations supporting its healthcare sector. Our data provide a clear, evidence‐based mandate for interventions that move beyond a purely clinical focus to address the profound socioeconomic and gender‐based determinants of health for cancer patients.

First, the overwhelming financial burden documented in this study must be recognized as a public health emergency. Policies aimed at mitigating this financial toxicity are paramount. This could include establishing national health funds, creating partnerships with international aid organizations to subsidize the cost of cancer diagnostics and treatment, and ensuring that essential cancer medicines are available and affordable in the public sector. Without addressing the catastrophic OOP expenditures, any efforts to improve clinical care will fail to reach the majority of the population.

Second, the stark gender disparity in QoL necessitates the development of gender‐sensitive cancer control programs. There is an urgent need to advocate for the reversal of policies that restrict women's access to education and healthcare. In the interim, practical strategies must be implemented to overcome existing barriers. This includes expanding community‐based screening programs staffed by female health workers, establishing mobile health clinics to reach women in rural areas, and creating safe and culturally appropriate transport mechanisms for women who need to travel for treatment.

Third, the significant symptom burden and its correlation with disease progression highlight the critical need to integrate palliative and supportive care into standard oncology practice. QoL assessment tools, such as the EORTC QLQ‐C30, should be incorporated into routine clinical workflows to systematically identify and manage distressing symptoms like pain, fatigue, and dyspnea from the time of diagnosis. Training healthcare providers in palliative care principles is a low‐cost, high‐impact intervention that can dramatically improve patient well‐being.

Finally, our findings underscore the importance of public health literacy. National awareness campaigns focused on the early signs and symptoms of common cancers are essential to combat the culture of late‐stage diagnosis. These campaigns must be culturally sensitive and utilize channels that are accessible to a population with high rates of illiteracy, such as radio broadcasts and community health worker outreach. By addressing these interconnected challenges, policymakers can begin to build a more equitable and effective cancer care system that improves not only survival rates but also the QOL for one of Afghanistan's most vulnerable populations.

## 5 Conclusion

This pioneering study in Afghanistan reveals the multifaceted and severe impact of cancer on patients' lives, where clinical progression is deeply intertwined with extreme financial hardship, social isolation, and gender inequality. The significant associations among cancer stage, sociodemographic factors, and QoL underscore the urgent need for holistic cancer care strategies that address these physical, emotional, and socioeconomic determinants. The findings emphasize the critical importance of integrating comprehensive QoL assessment into routine oncology practice to guide patient‐centered care. Future research should employ mixed methods and longitudinal designs to further explore the complex interactions between clinical, demographic, and psychosocial factors. By addressing the diverse needs identified in this study, healthcare providers and policymakers can enhance the overall well‐being of individuals undergoing cancer treatment, ultimately leading to better health outcomes and an improved QOL in this challenging context.

## Author Contributions

A.R., M.M., and E.E. proposed the idea and drafted the paper. E.E. joined the field study. A.R., M.M., and F.A.S. supervised the data collection. A.R. worked on the data analysis. M.J. and N.A.S. commented on the paper. A.R. and M.M. edited the paper. All authors read and approved the final manuscript.

## Funding

The authors have nothing to report.

## Ethics Statement

The study was conducted at the Oncology Unit of Herat Regional Hospital and received ethical approval from the Institutional Review Board of Jami University, which oversees research ethics and issues approval codes for registered proposals. Ethical clearance for this study was granted on January 27, 2024 (Ref: J.2024.1.27.1). All participants provided written informed consent prior to participation. For participants who were illiterate, informed consent was obtained through their legally authorized representatives (LARs). Participant confidentiality and privacy were maintained throughout the study in accordance with the Declaration of Helsinki and ethical principles guiding research involving human subjects.

## Consent

All participants provided written informed consent prior to participation.

## Conflicts of Interest

The authors declare no conflicts of interest.

## Data Availability

The data that support the findings of this study are available on request from the corresponding author. The data are not publicly available due to privacy or ethical restrictions.

## Endnotes

^1^
Tajiks are a Persian‐speaking ethnic group, predominantly Sunni Muslim, who constitute the second‐largest ethnicity in Afghanistan. In the context of Herat and western Afghanistan, they have deep historical and cultural roots. Unlike some other ethnic groups in Afghanistan, Tajik social structure is typically centered on village and family networks rather than formal tribal lineage, with loyalty patterns evolving around local community and kinship [20].
